# The splicing factor BRR2a functions as a repressor of stress-inducible genes

**DOI:** 10.1007/s44154-026-00345-x

**Published:** 2026-09-15

**Authors:** Jianfei Guo, Bangshing Wang, Huazhong Shi

**Affiliations:** Department of Chemistry and Biochemistry, Texas Tech University, Lubbock, TX 79409 USA

**Keywords:** Gene repression, DEAD-box RNA helicase, mRNA splicing, Abiotic stress, SHI6, BRR2a

## Abstract

Inducible gene expression is generally thought to be activated when transcription factors bind to promoter cis-elements, thereby recruiting the general transcriptional machinery to initiate gene transcription. However, the mechanisms that repress inducible genes under non-inducing conditions remain poorly understood. In this study, we identified a novel mutant, *shi6*, which exhibits slow growth, a dwarf phenotype, and elevated expression of a luciferase (LUC) reporter gene driven by a salt-inducible promoter. The *shi6* mutant also displayed increased sensitivity to abscisic acid (ABA), NaCl, and methyl jasmonate. Map-based cloning revealed that *SHI6* encodes the RNA helicase BRR2a, a core component required for pre-mRNA splicing and essential for plant embryogenesis. As expected, SHI6/BRR2a was required for the proper splicing of stress-inducible genes. Unexpectedly, SHI6/BRR2a also exhibited transcriptional activation activity in both yeast and Arabidopsis, suggesting that it associates with the transcriptional machinery and participates in gene transcription. Together, our findings demonstrate that SHI6/BRR2a is a dual-function protein that plays essential roles in both pre-mRNA splicing and the repression of stress-inducible genes under non-inducing conditions.

Being sessile, plants are constantly exposed to fluctuating natural environments and must respond rapidly to changes caused by biotic and abiotic factors. To cope with such changes, plants have evolved corresponding regulatory mechanisms. Among these mechanisms, alternative splicing (AS) has emerged as a regulatory step that enables plants to rapidly adjust gene expression without requiring de novo transcription (Alhabsi et al. 2025). Stress-induced AS reshapes the landscape of transcript isoforms, influences protein abundance and functional domain composition, and mRNA stability, thereby ensuring flexible and timely stress adaptation (Alhabsi et al. 2025). Specific splicing factors, including SR45a and RS40/RS41, have been shown to modulate abiotic stress responses, with their loss-of-function or overexpression leading to altered stress sensitivity (Xiong et al. 2026). Numerous evidence suggests that RNA helicases (RHs) regulate alternative splicing to fine-tune plant responses to different biotic and abiotic stresses (Nidumukkala et al. 2019; Pandey et al. 2020). However, the role of splicing factors in gene repression under normal growth conditions and rapid induction in response to environmental changes is still elusive. Here, we show the functions of SHI6/BRR2a, a DEAD-box RNA helicase crucial for mRNA splicing and essential for plant development, in the repression of stress-inducible gene expression under non-stress conditions. Our results indicate that, in addition to its role in pre-mRNA splicing, SHI6/BRR2a plays an important role in the control of stress-responsive genes and modulation of cold and phytohormonal responses in Arabidopsis.

To identify regulators of stress inducible genes, we employed a forward genetics approach to screen for mutations that alter the expression of luciferase (LUC) reporter gene under the control of salt-inducible *AtSOT12* promoter. Several mutants, designated as *shiny* (*shi*) mutants due to high LUC expression in these mutants, were isolated, and molecular characterizations of *SHI1*, *SHI2*, *SHI4*, *SHI5* have been previously reported (Jiang et al. 2013; Wang et al. 2017, 2020; Wu et al. 2021). Here we report on the molecular identity and functional analysis of *SHI6*. LUC imaging and quantitative measurements showed that *shi6* mutant seedlings exhibited clearly higher LUC activity than WT under both normal and stress conditions (Fig. 1A, B), indicating that SHI6 is a negative regulator of *SOT12P-LUC* expression. Northern blot analysis further revealed that the increased LUC activity in *shi6* was likely due to the increase in *LUC* transcript level, especially after salt stress treatments (Fig. 1C). Consistently, the transcript levels of native *SOT12* gene were also elevated in *shi6* mutant when compared with WT after salt stress treatments (Fig. 1C), which suggests that the transcriptional regulation of both *LUC* and *SOT12* is likely through the modulation of *SOT12* promoter. The *shi6* mutant displayed delayed growth and small stature indicative of growth and developmental defects under normal conditions (Fig. 1D). Extensive phenotyping of *shi6* mutant indicated that the *shi6* mutation resulted in hypersensitivity to ABA in seed germination (Fig. 2A, B) and seedling growth (Fig. 2C), and the *shi6* mutant exhibited more sensitive phenotype to MeJa and NaCl treatments compared with WT (Fig. 2C). Furthermore, the *shi6* mutant showed more severe growth inhibition in soil and agar plate under low-temperature stress compared with WT (Fig. 2D).

**Fig. 1:**
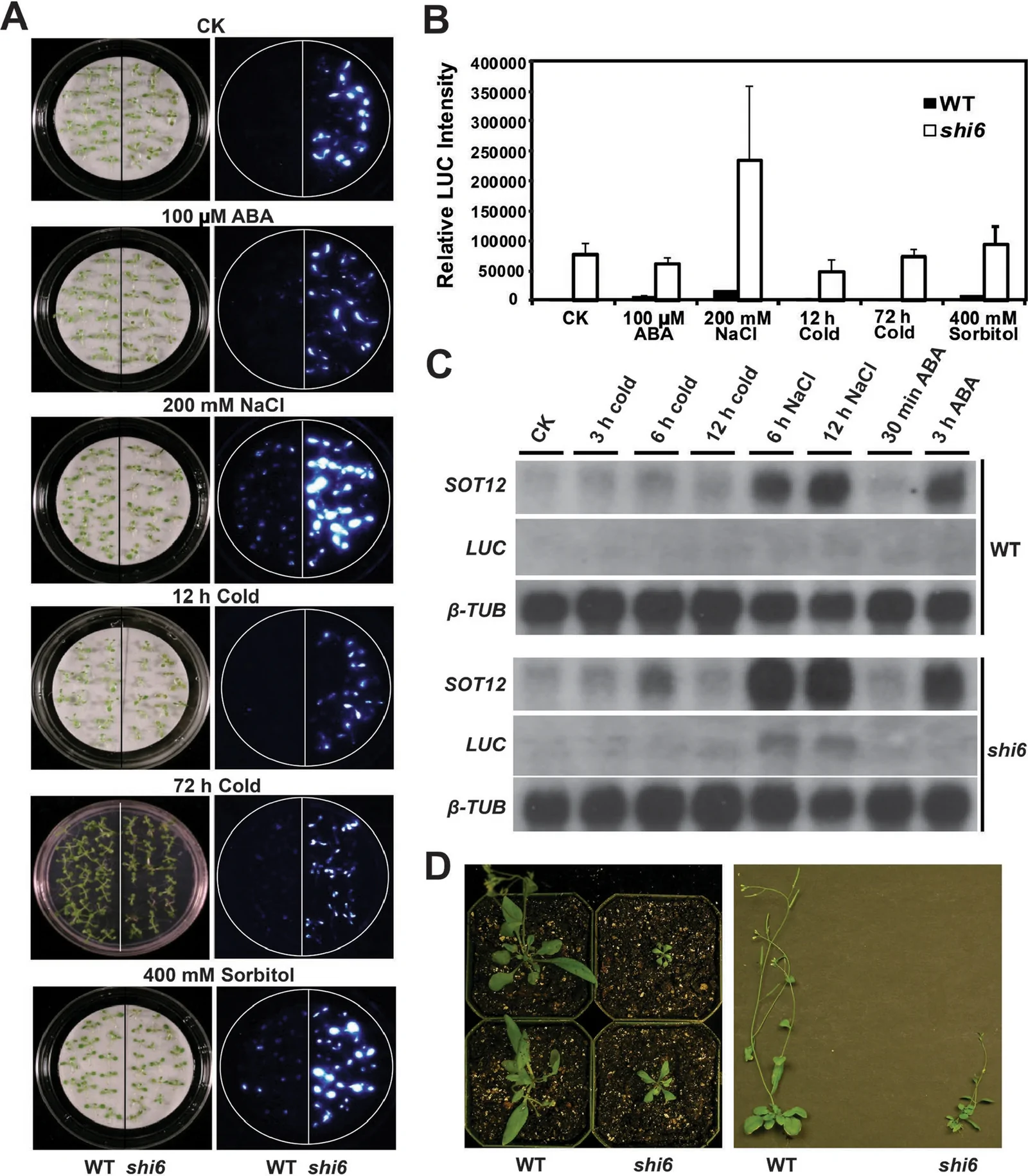
Identification and characterization of the *shi6* mutant. **A** Bioluminescence images of the wild type (WT) and *shi6* mutant under different stress conditions. 7-day-old seedlings grown on ½ MS medium were transferred to different stress conditions: 100 µM ABA for 3 h; 200 mM NaCl for 5 h; 400 mM sorbitol for 5 h; and 4 ℃ cold for 12 h and 72 h. The left panel shows bright field images, and the right panel shows bioluminescence images indicative of LUC expression levels. **B** Quantification of bioluminescence intensities in WT and *shi6* seedlings with or without stress treatments. Data are means ± SD (*n* = 10). **C** Northern blot showing the transcript levels of *LUC* and *SOT12* genes in WT and *shi6* seedlings under different stress treatments: 4 ℃ for 3, 6 h and 12 h; 100 mM NaCl for 6 and 12 h; 100 µM ABA for 30 min and 3 h. *β-TUB* was used as a loading control**. D** Morphological growth phenotype of *shi6*. Throughout plant development, *shi6* displayed slow growth and dwarf phenotype compared with WT and the leaves of *shi6* were more serrated than WT

**Fig. 2:**
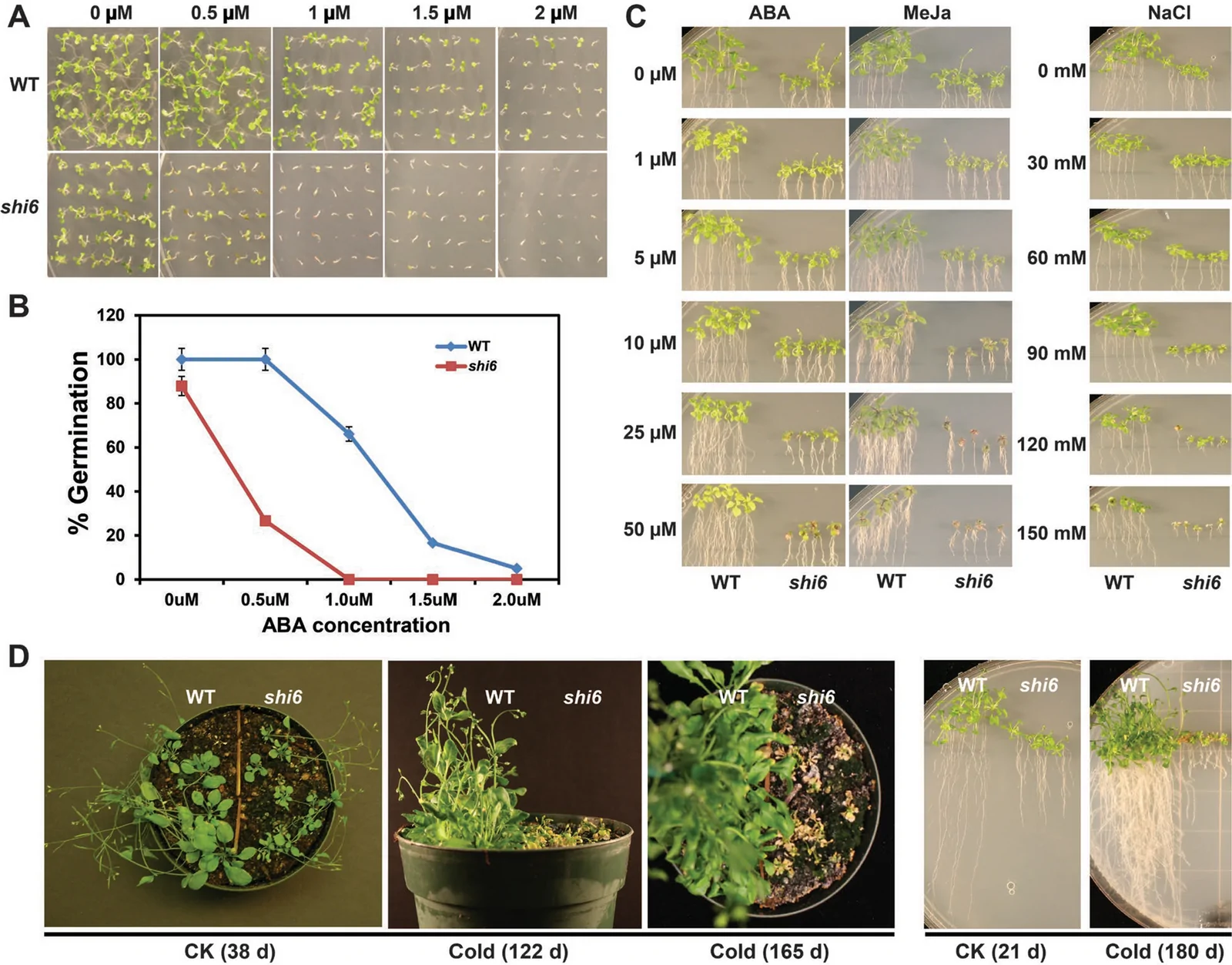
The *shi6* mutant is hypersensitive to exogenous phytohormones and abiotic stresses. **A** Seed germination of the WT and *shi6* mutant in ½ MS medium containing different ABA concentrations. **B** Germination rate scored with the formation of green cotyledons at the 6th day after growing on the medium. Data are means ± SD (*n* > 55). **C** *Shi6* seedlings displayed sick leaf growth phenotypes compared to WT under ABA, MeJa and NaCl treatments with indicated concentrations. **D** The *shi6* shows more severe growth inhibition under cold conditions than WT. Left panel shows growth phenotype in pots, and right panel shows growth phenotype in agar plate. 7-day-old seedlings from agar plates were transferred to pots and allowed for additional growth for 14 days under room temperature. These plants were placed under normal growth condition or in a 4 ℃ cold room for 180 d with continuous light

To determine the molecular identity of *SHI6*, we employed map-based cloning to identify the *shi6* mutation. The *SHI6* locus was mapped to the upper arm of chromosome 1 between the CER453463 (SSLP) and CER475270 (CAPS) markers. Sequencing analysis of candidate genes identified a single nucleotide substitution of G to A at 141 nt downstream of the translation start codon ATG, which resulted in a change of tryptophan to a nonsense codon in the gene *BRR2A* (At1G20960) (Fig. 3A). *BRR2A* gene is known to be required for embryo development since homozygous T-DNA mutants are embryo lethal (Tzafrir et al. 2004). To validate that *BRR2A* was indeed the *SHI6* gene, we selected two T-DNA lines (*emb1507-1* and *emb1507-2*) with insertion in the ORF region of At1G20960 gene and conducted genetic complementation tests between *shi6* and the heterozygous T-DNA lines. A 1:1 segregation ratio of mutant to WT in the F_1_ progeny was expected and was confirmed by the growth phenotype and LUC imaging; approximately half of the F_1_ seedlings displayed small stature with elevated LUC activity (Fig. 3B). To further confirm the LUC phenotype, the small and large F_1_ seedlings were separately transferred to a new agar plate and LUC imaging re-confirmed that the small F_1_ seedlings but not the large seedlings resembled the *shi6* LUC imaging phenotype (Fig. 3C, D). Genotyping of the small and large seedlings by PCR and Sanger sequencing indicated that the genetically complemented small seedlings possess both T-DNA insertion and the G to A substitution, while the non-complemented WT-like large seedlings only have the *shi6* mutation but no T-DNA insertion in the gene At1G20960 (Fig. 3D), further confirming that the T-DNA line is allelic to the *shi6* mutant. These results indicate that the G to A substitution in *BRR2A* is the causal mutation for the *shi6* mutant phenotypes, and the *shi6* mutation is a weak allele.

**Fig. 3:**
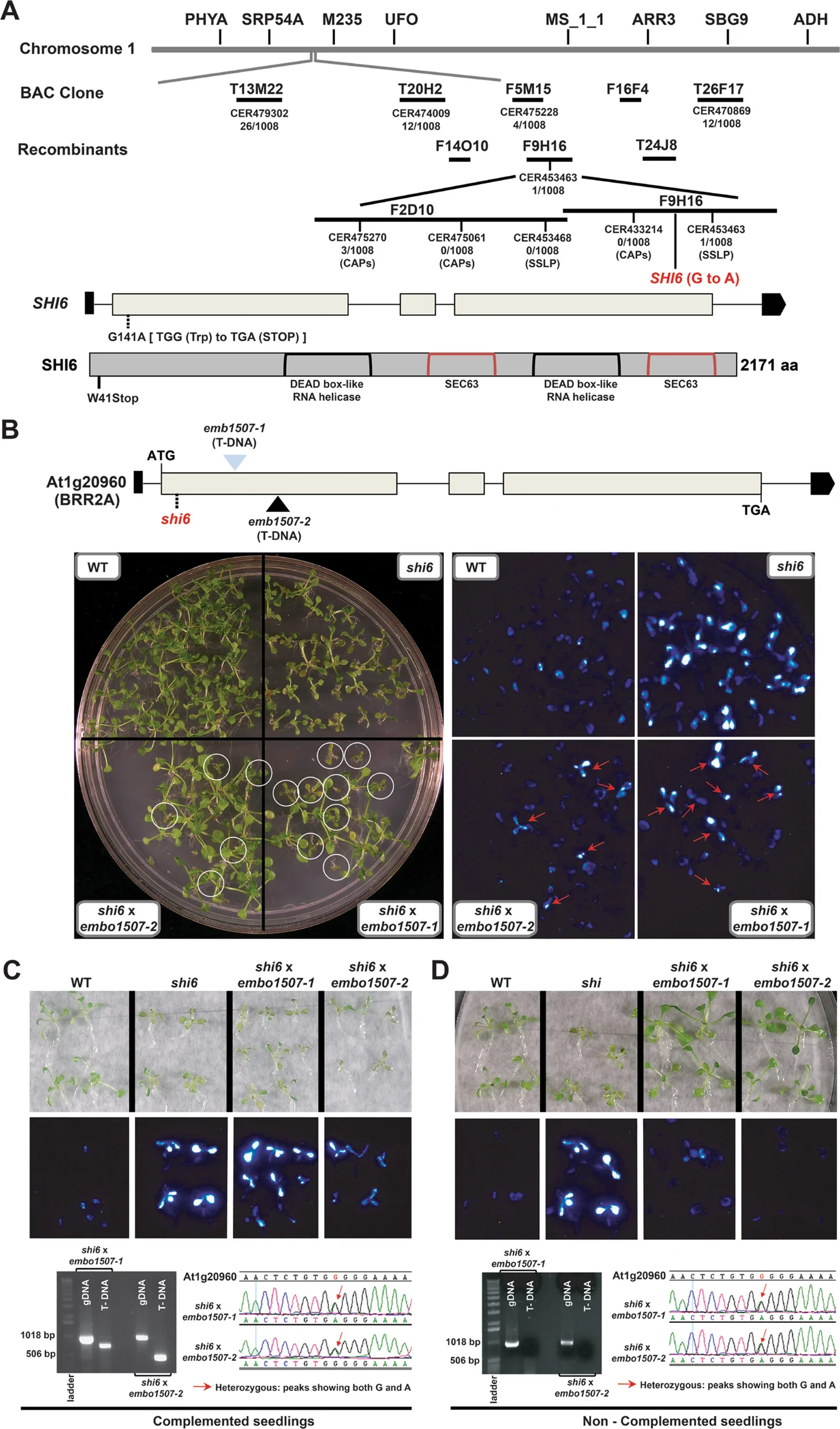
Map-based cloning and genetic complementation of the *shi6* mutant. **A** Map-based cloning of the *SHI6* locus. The *shi6* mutation is a G to A change at nucleotide position 141, resulting in a nonsense stop codon formation. **B** Genetic complementation of the *shi6* mutant with the T-DNA insertion mutants of *At1g20960* gene. The upper panel shows the gene structure of *SHI6* with the *shi6* mutation and T-DNA insertions indicated. The lower panel shows bioluminescence phenotypes of the F_1_ seedlings from the crosses between *shi6* and heterozygous T-DNA mutants (*embo1507-1* and *embo1507-2*). White circles indicate the F_1_ seedlings with small stature and red arrows show elevated LUC activity in these seedlings. **C** and** D** *shi6*-like smaller seedlings (C) and WT-like large seedlings (D) were separately imaged to re-confirm the luciferase phenotype and the mutations in the F_1_ seedlings. The upper panel shows bright field images and bioluminescence images. The lower panel shows the presence or absence of T-DNA insertions and the *shi6* mutation in the complemented small F_1_ seedlings and non-complemented large F_1_ seedlings by PCR-based genotyping and Sanger sequencing

BRR2 is well-known for its function in rearrangement of splicing complex during pre-mRNA splicing in various organisms and is essential for plant embryogenesis (Zhang et al. 2015; Absmeier et al. 2016; Li et al. 2023). To understand the role of *SHI6* in stress-responsive gene regulation, we determined whether *SHI6* is also involved in the regulation of other stress-responsive genes. Northern blot analysis showed that the expressions of *CBF1* and *CBF3*, but not *CBF2*, were clearly reduced in *shi6* than in WT under cold stress conditions, while the expressions of other abiotic stress responsive genes *COR15A*, *COR47*, *KIN1* and *DREB2A* were mostly similar between *shi6* and WT (Fig. 4A). However, slight reduction in the expression of *COR15A* and *COR47* in *shi6* at 30 min by ABA treatment was observed (Fig. 4A). Intriguingly, we found additional bands with slightly larger size of the *COR15A* and *KIN1* transcripts after cold treatments (Fig. 4A), suggesting possible mis-splicing of pre-mRNA in the *shi6* mutant. Detection of intron retention in *COR15A* and *KIN1* transcripts showed that the *shi6* mutant accumulated substantially more intron-retaining mRNAs than WT under cold stress conditions. Higher accumulation of intron-containing mRNA *in shi6* was further confirmed via Northern blotting using both *COR15A* coding and intron regions as probes (Fig. 4C). Since the *shi6* mutation is a weak allele (null alleles are lethal), the mRNA splicing in *shi6* could be affected at a low extent. Nonetheless, our results confirmed that SHI6 is required for proper mRNA splicing of some of the cold responsive genes, which is consistent with previous reports showing that BRR2a activity was required for proper splicing of a small group of introns in flowering time and miRNA biogenesis (Mahrez et al. 2016; Li et al. 2024).

**Fig. 4:**
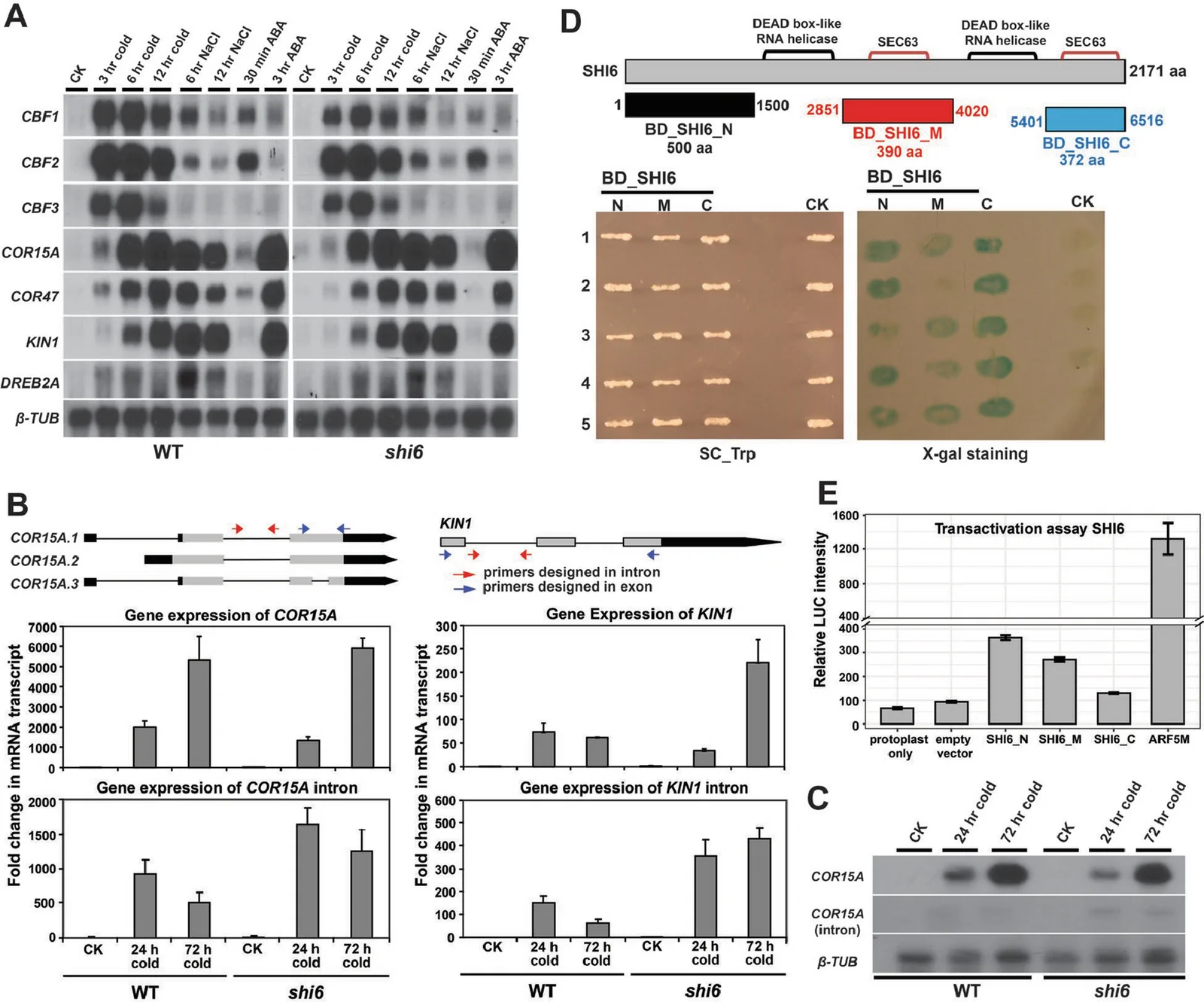
SHI6 mediates alternative splicing and transcriptional activation. **A** Northern blot showing the transcript levels of stress-inducible genes in WT and *shi6* in response to different stress treatments. *β-TUB* was used as a loading control. **B** Detection of intron retention in WT and *shi6* mutant. The top panel shows gene structure of *COR15A* and *KIN1* in Arabidopsis. The bottom panel shows gene expression levels quantified by RT-qPCR. 7-day-old seedlings grown under normal conditions were treated without (CK) or with 4 ℃ for indicated time. Primers from both coding and non-coding regions were used to study the expression levels of *COR15A* and *KIN1*, and intron-containing *COR15A* and *KIN1* in WT and *shi6*. Data are means ± SD (*n* = 3). **C** Gene expression of intron-containing *COR15A* analyzed by Northen blotting. Total RNA (20 µg) from 7-day-old seedlings from different cold treatments were subjected to RNA hybridization with probes from coding and non-coding regions of *COR15A*. *β-TUB* was used as a loading control**. D** Transcriptional activation of *β-Gal* gene by SHI6_N, SHI6_M and SHI6_C in yeast. Different forms of SHI6 were fused with the DNA-bind domain (BD) in the bait construct, and self-activation assay was used to determine the transcription activation activity. Empty vector (CK) was used as a negative control. Rows 1–5 indicate five independently transformed colonies analyzed. **E** Transcriptional gene activation assay using Arabidopsis protoplasts. *ARF5M* (Auxin Response Factor 5 Middle region) was used as a positive control. Data are means ± SD (*n* = 3)

BRR2a, BRR2b and BRR2c are three unique RNA helicases in Arabidopsis that have a tandem repeat of DEAD box-like RNA helicase domain with extraordinary molecular mass (more than 2,000 amino acids (aa)), but only *BRR2a* showed ubiquitous expression (Li et al. 2023). The overall structure of SHI6/BRR2a protein is made up of a long N-terminal region containing about 500 aa followed by the first DEAD box-like RNA helicase from 514 to 653 aa. In between the two tandem connected DEAD box-like RNA helicase is a known domain SEC63. The second DEAD box-like RNA helicase is positioned from 1,360 to 1,537 aa followed by another SEC63 domain which completes the C-terminal region of SHI6 (Fig. 4D). Removal of the entire N-terminal domain of BRR2 is lethal to *Saccharomyces cerevisiae* and deletion of the N-terminal 120 residues does not significantly affect Brr2's helicase activity but leads to splicing defects and severely impaired growth (Zhang et al. 2015). Li et al. demonstrated that Brr2a is able to directly bind to pri-miRNAs in vivo, and truncated BRR2a (N-Brr2a [487–1,288 aa], C-Brr2a [1,289–2,171 aa]) and its variants (N-EQ [E640Q] or N-AA [S676A and T678A]) are capable of binding dsRNA, and N-Brr2a exhibited higher binding affinity to folded pri-miR159b (Li et al. 2024). To test which part of the Arabidopsis SHI6/BRR2a transcriptionally regulates gene expression, we fused different regions of SHI6 with a DNA-binding domain in the yeast bait construct. Interestingly, all of the N-terminal (SHI6_N [1–500 aa]), middle (SHI6_M [950–1,340 aa]) and C-terminal (SHI6_C [1,800–2,172 aa]) regions of SHI6 activated the reporter gene (β-*Gal*) expression in yeast cells (Fig. 4D). These results suggest that SHI6 can be associated with the transcriptional machinery to modulate gene transcription in yeast. To further confirm the function of SHI6 in transcriptional gene regulation in plants, transcription activation assays were performed in plant cells as described previously (Tiwari et al. 2003; Yoo et al. 2007). The protoplasts with SHI6_N, SHI6_M, or SHI6_C fusion proteins displayed significantly higher LUC activity than those with the empty vector. And the SHI6_N fusion protein showed the highest transcriptional activation activity, although lower than the known activator ARF5M as a positive control (Fig. 4E). These results further support that SHI6 could function as a transcriptional regulator in plant cells. Together with the stress response phenotypes and intron-retention that *shi6* displayed, *SHI6* is proposed to be a dual functional protein playing important roles in mRNA splicing and repression of stress-inducible genes. Its repression function could be independent of its splicing function because both *LUC* gene and *AtSOT12* gene that are repressed by SHI6/BRR2a are intronless genes.

In conclusion, we have identified SHI6 as a repressor for salt-inducible genes. SHI6 is also involved in maintaining proper mRNA splicing of cold-responsive genes. Using this powerful forward genetic mutant screen system, we identified several SHI proteins that are important in stress-inducible gene regulation. SHI1 and SHI4/CPL1 form a complex that represses stress-inducible gene transcription by preventing mRNA capping and transition from transcription initiation to elongation (Jiang et al. 2013), SHI5/HDA6 inhibits defense response to pathogens via repressing the expression of pathogen-inducible genes (Wang et al. 2017), and previous studies showed that SHI2 may function as a component in a ready-for-transcription repressor complex to regulate gene repression and activation under normal and stress conditions, respectively (Wang et al. 2020). Together with the findings in this study, we propose that under normal growth conditions, the inducible promoters, including the *AtSOT12* promoter used in this study, could be occupied by a repression complex. This complex may contain the general RNA polymerase II transcription machinery, SHI1 and SHI4, SHI5/HDA6 histone deacetylase, and co-transcriptional splicing factors SHI2 and SHI6 to form a ready-for-transcription repressor complex. Under stress conditions, activation of the inducible genes could be triggered by changes of the components in the complex, leading to de-repression and rapid activation of gene transcription without the need of assembling the transcription and co-transcription proteins at the inducible promoters. Although this hypothesis requires extensive experimental validation, our findings suggest that SHI6 is likely a repressor associated with the transcription initiation machinery to inhibit stress-inducible gene transcription under normal conditions.

## Materials and methods

### Plant materials and growth conditions

In this study, the Arabidopsis (*Arabidopsis thaliana*) accession Col-0 and *Landsberg erecta* (Ler) were used. The background line containing *SOT12:LUC* transgene, EMS mutagenesis, and isolation of the *shi* mutants were described in our previous study (Jiang et al. 2013). The T-DNA insertion mutant lines *emb1507-1* and *emb1507-2* were obtained from the Arabidopsis Biological Resource Center (ABRC). The heterozygous mutants of the T-DNA lines were identified by PCR-based genotyping and used for genetic complementation. For all plate-based assays, the surface-sterilized and stratified (4 ℃ for 48 h) seeds were sown on 0.5 × Murashige and Skoog medium (pH5.8) containing 1% (w/v) sucrose and 0.6% (horizontal) or 1% (vertical) (w/v) agar and grown in a growth chamber set to normal growth conditions with 22 ℃ and 16 h/8 h light to dark cycle. The stress treatment conditions were specified in the figure legends.

### Luminescence assay

The 7-day-old seedlings grown on ½ MS (Murashige and Skoog salt base; JRH Biosciences, Lenexa, KS) agar plates were treated with different types of stresses and then subjected to LUC imaging by using the Andor CCD luciferase imaging camera (DU434-BV, Andor Technology, Connecticut). ABA, NaCl, sorbitol, and cold treatments were conducted by incubating seedlings on filter paper saturated with ½ MS liquid media containing 100 µM ABA for 3 h, 200 mM NaCl for 5 h, 400 mM Sorbitol for 5 h, or at 4 ℃ for 12 h and 72 h, respectively. Luminescence imaging was conducted by using the Andor CCD imaging system and quantification of relative luminescence intensity in each seedling was performed by using the MCD software equipped with the imaging system.

### Northern blot analysis

Northern blot analysis was conducted by following our previously described method (Chung et al. 2008). In brief, control and stress-treated 7-day-old seedlings were collected for RNA extraction using the extraction buffer containing 50 mM Tris–HCl pH at 8.0, 300 mM NaCl, 5 mM EDTA pH at 8.0, 2% SDS, 2 mM aurintricarboxylic acid (ATA), and 10 mM β-mercaptoethanol as previously described (Shi and Bressan 2006). Total RNAs were separated into a 1.3% agarose gel and transferred to nylon membrane using capillary blotting system. The abundances of gene transcripts were then determined by hybridization with corresponding ^32^P-labeled probes and X-ray film imaging.

### Map-based cloning and genetic complementation

The *shi6* mutant was crossed with Ler ecotype, and the resulting F_1_ was self-pollinated to generate F_2_ progeny. The LUC expression levels of 5-day-old F_2_ seedlings were evaluated by LUC imaging and individuals with higher luminescence were selected and used for map-based cloning of the *SHI6* gene. For genetic complementation, the *shi6* mutant was crossed with the heterozygous T-DNA mutant alleles, and the F_1_ seedlings were subjected to LUC imaging. The presence of T-DNA insertion and the *shi6* mutation was determined by PCR-based genotyping and Sanger sequencing, respectively.

### Detection of intron retention by RT-qPCR

RT-qPCR was used for detection of intron retention. For RT-qPCR, 7-day-old seedlings of the WT and *shi6* mutant grown on ½ MS medium under normal conditions (control) were used for cold treatment. Total RNAs were isolated from control and cold-treated seedlings using the Plant RNA Purification Reagent (Invitrogen). cDNA was synthesized by using the AMV reverse transcriptase (Promega) and then used as templates for quantitative PCR using ABI PRISM 7500 Real-Time PCR Systems (Applied Biosystems) and the iTaq SYBR Green Supermix with ROX kit (Bio-Rad, Hercules, CA, USA). To determine the intron retention in RNA samples, primers were designed to specifically amplify the sequences of introns or the sequences of exons of the target genes, as indicated in the figure, *ACT2* gene was used as an internal control. All the reactions were performed in triplicate (Wang et al. 2020).

### Transcription activation assay

Self-activation tests were performed using different portions of the SHI6. The N-terminal (N), middle (M), and C-terminal (C) of *SHI6* cDNA sequence were amplified by PCR. The amplified sequences were cloned into the pENTR1A vector and then recombined into the destination vector pGBKT7. The resulting constructs were then transformed into yeast strain Y190. Yeast cells grown on a synthetic medium without tryptophan were subjected to β-galactosidase assay. The effector-reporter system used in this study was described previously (Chinnusamy et al. 2003). The coding sequence for the SHI6_N protein from amino acids 1 to 500, for SHI6_M protein from amino acid 951 to 1,340, and SHI6_C protein from amino acids 1,800 to 2,172 was cloned into the effector vector (35S-GAL4 BD-NOS), respectively. Then both effector and reporter (5XGAL4-TATA-LUC-NOS) plasmids were co-transformed into Arabidopsis protoplasts and LUC activity was determined by following the methods described by Yoo et al. (Yoo et al. 2007).

## Data Availability

The materials used in this study will be available for research upon request.

## Abbreviations

ABA: Abscisic acid
AS: Alternative splicing
BRR2a: RNA helicase BRR2 homolog A
BRR2b: RNA helicase BRR2 homolog B
BRR2c: RNA helicase BRR2 homolog C
CAPS: Cleaved amplified polymorphic sequence
CBF: C-repeat-binding factor
COR15A: Cold-regulated 15a
COR47: Cold-regulated 47
DREB2A: Dehydration-responsive element-binding protein 2A
DEAD-box protein: Motif Asp-Glu-Ala-Asp contained protein
KIN1: Stress-induced protein KIN1
LUC: Luciferase
ORF: Open reading frame
RHs: RNA helicases
RS40/RS41: Serine/Arginine factor SR40/41
SHI1: K-homology domain containing protein SHINY1
SHI2: DEAD-box RNA helicase SHINY 2
SHI4/CPL1: SHINY4 / CTD phosphatase-like 1
SHI5/HDA6: SHINY 5 / Histone deacetylase 6
SHI6: DEAD-box RNA helicase SHINY 6
SOT12: Sulfotransferase 12
SR45a: Serine/Arginine factor SR45a
SSLP: Simple sequence length polymorphism
